# Improvement of trip generation rates for mixed-use development in Klang Valley, Malaysia

**DOI:** 10.1038/s41598-023-29748-w

**Published:** 2023-02-15

**Authors:** Boon Hoe Goh, Choon Wah Yuen, Chiu Chuen Onn

**Affiliations:** 1 Department of Civil Engineering, Faculty of Science and Engineering, University of Nottingham Malaysia, 43500 Semenyih, Malaysia; 2 Centre for Transportation Research, Department of Civil Engineering, Faculty of Engineering, Universiti Malaya, 50603 Kuala Lumpur, Malaysia

**Keywords:** Civil engineering, Statistics

## Abstract

Mixed-use developments (MXDs) are a single development project that integrates and interacts with different land uses. Traffic is estimated to be reduced with such development. However, there is no standard procedure is available to estimate the MXD trip generation rates in Malaysia. The Malaysian Trip Generation Manual (MTGM) is the guidelines currently been used to forecast future trips for single land use. If the trip generation rates for multiple land uses are summed as MXD trips, the total trips will be overestimated without considering the internal trip capture. This study aimed to establish an improved method for estimating the MXD trip generation rates. Four MXD observation sites were selected in Klang Valley. Traffic survey counts were conducted considering person-trip, including passengers in vehicles and pedestrians. The results revealed that the MXD trip generation rates with MTGM were higher than actual traffic counts during peak hours. The MXD adjustment factor was established as 0.63, which can be applied by multiplying the MTGM trip generation rates to reduce the generated MXD trips in PCU per hour. This research has formulated a new data collection method by integrating person counts, alongside with new guidelines for pedestrian counts. The findings provide an option to adjust the MXD trips and prevent from overestimating future trips, which may result in overdevelopment and spending on mitigation measures in urban planning and road infrastructure.

## Introduction

Traffic engineers and transport planners often face challenges in estimating the vehicular trips for a single-use or mixed-use development (MXD) project during traffic impact assessment (TIA). The MXDs are a single development project that integrates and interacts with different land uses, such as offices, retail, restaurants, entertainment, hotels, or residential. On the other hand, TIA is a traffic study to evaluate the traffic condition by employing a level of service (LOS), before-and-after the proposed development. To begin with, TIA undergoes a sophisticated process and complicated transportation demand modelling (TDM) to identify traffic impacts due to new development and mitigation measures. Therefore, ensuring that mitigations are adequate but not excessive is essential by first looking at the trip generation model for future trips. Overestimating future trips may result in overdevelopment and spending on mitigation measures. The MXD is the new development concept trend, and limited studies have been conducted to estimate trip rates. The common practice for MXD trip generation studies is to combine trip rates of different types of land uses. Unfortunately, such a method potentially overestimates the actual trip generation rates for MXD. In addition, the current trip generation manuals present several limitations to support the MXD trip generation study and should be appropriately addressed to match the current practice.

The Malaysian Trip Generation Manual (MTGM) 2010^1^ was published by the Highway Planning Unit (HPU), Ministry of Works Malaysia. The MTGM has also been named as HPU’s trip generation manual, providing trip generation information on sixty-one (61) different land-use types in Malaysia. The MTGM estimates the number of trips as a function of the type of the development (land use type) and correlates these estimations with the standard and easily measurable parameters that usually represent the sites’ characteristics, such as square footage (GFA), number of dwelling units, and number of filling points at a petrol station. The MTGM is referenced and based on the Institute of Transportation Engineers (ITE) Trip Generation Manual, especially in establishing the trip generation rates as weighted average trip rates and regression analysis.

With the limited types of land for single-use and MXDs, many local researchers have raised their concerns and conducted appropriate studies to complement the trip generation rates. Presently, the manual is only limited to estimating single land use and is not suitable for estimating vehicular traffic at MXDs. Hence, the actual traffic may be overestimated. The ITE’s trip generation manual is more reliable and comprehensive, which considering internal trips capture for estimating the trip generated by MXD. Nevertheless, this manual cannot be adopted directly because the western conditions may not apply to Asian cities, such as Malaysia.

The trip generation studies undertaken by the HPU are still limited and failed to integrate the MXD and transit-oriented development (TOD) into the trip estimation. Researchers in Malaysia, such as Anil et al.^2^ and Ishtiaque et al.^3–5^, addressed the limitations of MTGM and intended to overcome the weaknesses by undertaking trip generation studies for specific land use. The hypermarket trip generation study by Anil et al.^2^ in Johor Bahru revealed that ITE and HPU trip generation manuals provide trip rates insensitive to population density, travel patterns, economic growth, accessibility, and others. Thus, these trip rate calculation procedures are prone to biases or errors, resulting in either an overestimation or underestimation. Anil et al.^2^ suggested including the socio-economic, demographic and land use data as critical variables for trip rates estimation.

According to Ishtiaque et al.’s findings^3^ in a fast-food restaurant trip generation study, the HPU’s manual provides a trip generation relationship with GFA. In contrast, ITE’s manual offers trip generation data based on three different parameters: GFA, number of seats, and peak hour traffic on the adjacent street. The study showed that the GFA and number of parking spaces were significant parameters in determining the number of trip generations. Nonetheless, no clear relationship could be established between trips and the number of seats. The trip generation rates of the study based on GFA were higher than HPU’s manual due to rapid urbanisation and the increased population growth and vehicle ownership in Johor Bahru.

Another similar study was undertaken by Ishtiaque et al.^4^ also reported that the HPU’s manual does not provide any coefficient of determination value (R^2^) or line of best fit for land use type of polyclinics. The independent variable (GFA) is unable to differentiate between big-scale hospitals and small clinics, which were grouped in the same category. The study revealed that the HPU’s manual underestimates the number of trips generated by the polyclinic land-use type in Johor Bahru. Thus, the GFA is not the best parameter for estimating the trips generated by polyclinics in Malaysia. This limitation might be one of the reasons why polyclinic trip generation rates in HPU’s manual are lacking in the line of the best fit and show weak correlations with small R^2^ values. Out of the four independent variables considered, only the number of doctors was found to be the most significant in determining the number of trips generated.

Ishtiaque et al.^5^ identified that the land use type of kindergarten in the HPU trip generation manual based on the GFA parameter has three data points. The value indicates insufficient data points and an incomplete regression model for accurately estimating trip generation rates. Therefore, his team undertook a trip generation study for kindergarten land-use type in Johor Bahru by using different parameters such as number of students, number of staff, and number of schools within a 3 km radius. Nevertheless, the number of students was found to be statistically significant.

MTGM is a trip generation manual published mainly for estimating the single land-use of trip generation rates. This manual has few limitations as discussed and can be improved. One of the limitations is, the MTGM does not recommend any method to forecast the MXD trip generation rates and therefore, current practice is to estimate the MXD trip generation rates by combining multiple single land-use trip generation rates. Such approach is overestimating the MXD trip generation rates without considering the internal trips capture. This research aimed to establish new methods to estimate the MXD trip generation rates by identifying the adjustment factor to be used alongside MTGM.

## Single use and MXDs

Single-use development involves only one land used and real-estate development such as residential, commercial, or recreation development. Many countries adopted ITE’s Trip Generation Manual to estimate the trip rates and traffic impact due to the new development. Nonetheless, in Malaysia, traffic engineers and planners prefer to apply MTGM published by HPU, Ministry of Works Malaysia. Both manuals will be further discussed in the later section.

According to ITE^6,7^, MXD is defined as a single real-estate development that consists of land uses corresponding to two or more ITE land-use types between which trips can be made without the off-site road system. It may also be referred to as a multi-use development. In MXD, trips between various land uses can be made on-site, and these internal trips do not utilise the major street system. The internal trips can be made either by walking or by vehicles using internal roadways without external streets. On the other hand, an internal capture rate can generally be defined as the percentage of total person trips generated by a site made entirely within the site^6,7^.

As defined by ITE^6,7^, a traditional downtown or central business district (CBD) is not considered MXD. Downtown areas typically comprise a mixture of diverse employment, retail, residential, commercial, recreation and hotel use. Extensive pedestrian interaction occurs due to the scale of the downtown area, ease of access, and proximity of the various uses. Automobile occupancy is usually higher in the CBD than in outlying areas, particularly during peak commuting hours. Some downtowns have excellent transit service. For these reasons, trip generation characteristics in a downtown environment are different from a general urban or suburban area.

A shopping centre could also be considered an MXD because it typically includes uses other than general retail, such as restaurants, banks, and offices. Shopping centres are considered in ITE’s Trip Generation Manual as a single land use because data have been collected directly for them as stand-alone development. Conversely, if the shopping centre is part of a larger MXD or is planned to have out-parcel development of a significantly different land-use type, the site could be considered an MXD to estimate site trip generation^6,7^.

Transit-oriented development (TOD) is an MXD and pedestrian-friendly precincts around transit stations. It is an increasingly popular strategy for encouraging smart growth in Australia and the United States^8^. Smart growth is a comprehensive urban generated planning and transportation theory that imparts growth in a city’s centre to decrease the urban sprawl and creates compact, transit-oriented, walkable, bicycle-friendly land use, including neighbourhood schools, complete streets, and MXD with a vast range of housing choices^9^.

Communities are encouraging development that follows the principles of smart growth (higher densities, mixed land uses, and infill locations) as a strategy for reducing vehicle travel^9^. A subdivision or planned unit development containing general office buildings and support services such as banks, restaurants, and gasoline service stations arranged in a park-or-campus-like atmosphere should be considered an office park, not an MXD^6,7^. An office building with support retail or restaurant facilities inside the building should be treated as a general office building. A hotel with an on-site restaurant and small retail should not be treated as an MXD^6,7^.

## Methods

### Study location

Three sites were selected in Klang Valley of Selangor state, Malaysia. All sites were screened thoroughly with a list of criteria adopted for this study to ensure homogeneity and maturity for data collection. Table 1 shows the site selection criteria, a part of the checklist during the preliminary site visit. Only sites that fulfil these criteria were selected to ensure and maintain the consistency of the study objectives. Due to multiple reasons, including permission to reveal the actual name of the MXD sites, the selected MXD sites are generally described.

**Table 1:** Site selection criteria checklist.

No.	Characteristics of a mixed-use development site	Screening outcomes after the site visit
1	Is the site a single real estate project?	
2	Does the site consist of two (2) or more land-use types?	
3	Is the site fully developed/built?	
4	Does the built portion of the site have a reasonably high occupancy rate and/or appear to be economically healthy?	
5	Is the site mature (over two years old)?	
6	Can the trips between various land use components be made without using the off-site roadway system?	
7	Does the site have its own independent driveway(s) not shared with any adjacent property?	
8	Is the presence of through traffic minimal?	
9	Are the site’s internal and/or adjacent streets free of any construction activity that would interfere with the traffic flow?	
10	Is the site away from CBD/downtown area?	
Does the site meet mixed-use development criteria?		

The first site (Site A) of data collection is located at the Jalan USJ 21/10, USJ 21, 47,630 Subang Jaya, Selangor, Malaysia and has been open for service since 2014. This MXD is considered as two types of land use vertically integrated. The bottom part is a four-storey shopping mall, while the upper part is a four-block residential condominium. The second site (Site B) is a vertical MXD with retail shops beneath the residential apartment located at Desa Pandan, Off Jalan Desa Pandan, 55100, Kuala Lumpur, Malaysia. This MXD was completed in 2015 and has been established for more than four years. The third site (Site C) is an MXD of horizontally integrated luxury condominiums and retail shops. It is located at the Jalan Persiaran Tropicana, Tropicana, 47410 Petaling Jaya, Selangor, Malaysia. It was completed in 2008 and consisted of five blocks. Each of the blocks is different in terms of residential dwelling units. Only the ground and first floors of one residential block are allocated for commercial purposes, while the other blocks are purely residential. Several internal corridors have been built between each block to allow residences easy accessibility and only require a few minutes of walking. All the selected sites have two land uses only, and the occupancy rate was more than 80%.

The traffic survey conducted at these three sites was primarily for modelling and computation adjustment factors. For the purpose of validating the models and adjustment factors, another site, namely Site D, was selected for traffic survey but with fewer details. Only the vehicular volume count was determined during peak hours on weekdays and weekends. The fourth site (Site D) is situated in Danau Kota, a township in Setapak located along Jalan Genting Klang in Kuala Lumpur, Malaysia. This MXD site has been in service since 2011 and with a shopping mall and condominium integrated vertically.

### Traffic study

The traffic study was divided into two stages: traffic count survey and desktop study. The primary data was obtained by traffic counts for both vehicular and pedestrians at the major entrances and exits during weekdays and weekends at the selected MXD sites. Before the actual data collection, a pilot study was conducted to identify the sites’ conditions. A trial survey was undertaken to identify the ideal survey spots and potential hazards and access the anticipated research challenges during actual data collection. Nevertheless, the secondary data was compiled through a desktop study and site observations. Drawings and data such as floor plan, number of parking lots, the number of dwelling units for office or residential units, and gross floor area (GFA) were collected. This information was helpful in the estimation of trips generated using the MTGM and forecasting model development.

**Figure 1:**
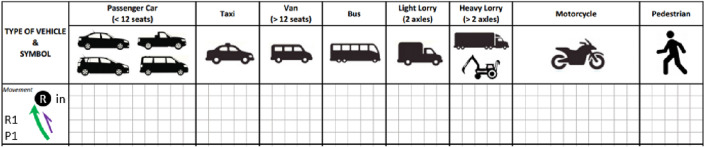
Traffic survey form.

The research aimed to integrate person-trips into the trip generation model. Hence, a new data collection procedure was developed exclusively. The data collection form was designated to record the vehicle, person, and travel modes data, respectively. Figure 1 shows the traffic survey form where person trip by travel modes data can be collected. This research was primarily focused on the trip generation base on their land use. Hence, each site’s ingress and egress traffic volumes have to be recorded and classified into eight (8) categories: Passenger Car, Taxi, Van, Bus, Light Lorry, Heavy Lorry, Motorcycle, and Pedestrian. The occupancy rate of the vehicles was recorded by counting the number of people (driver and passenger) in the vehicle. Each cell in the form represents one vehicle unit with a written number of persons.

**Figure 2:**
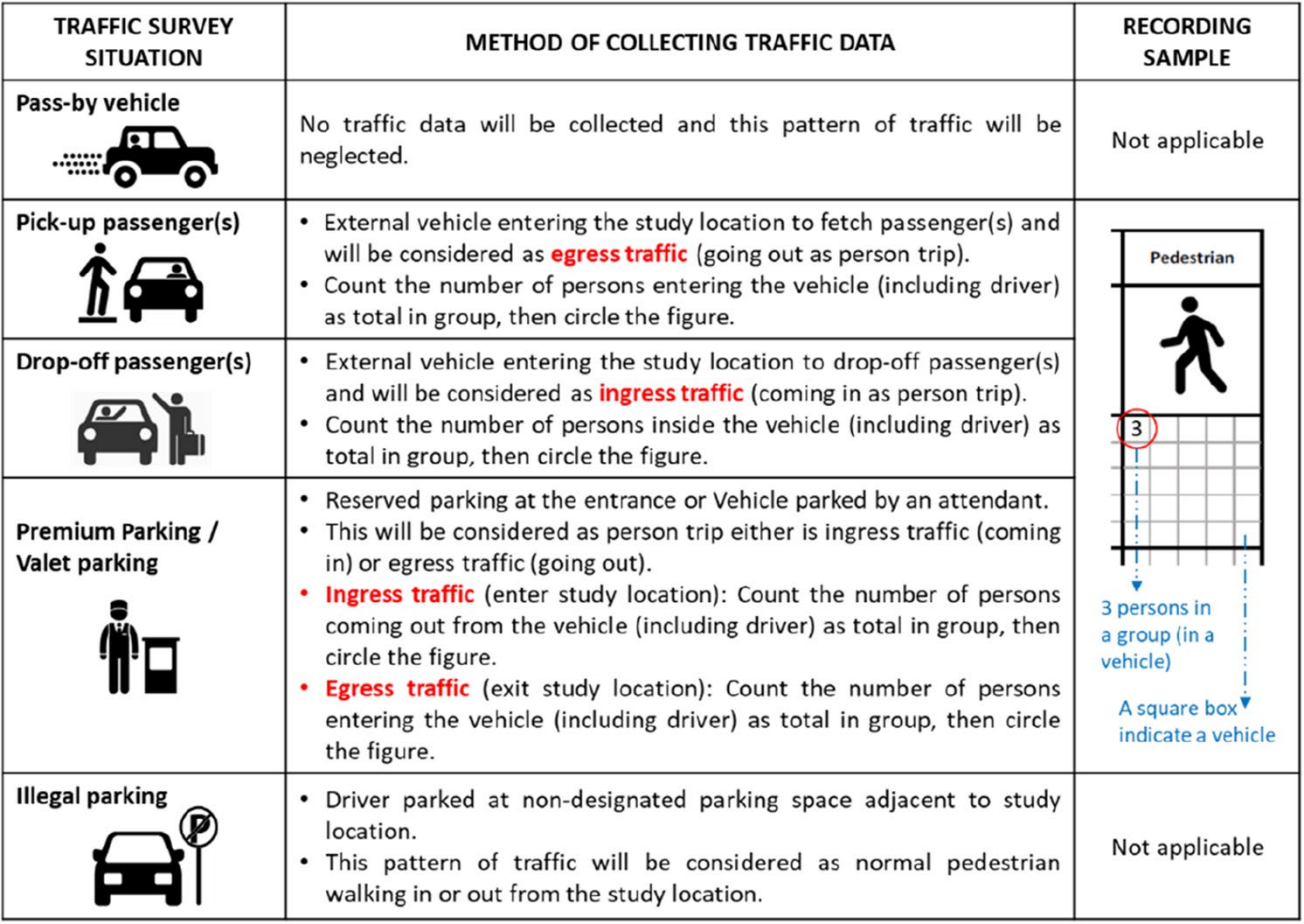
Guidelines for traffic survey (Pedestrian count).

Nevertheless, a guideline was developed as part of the new data collection procedure for the person or pedestrian count. Figure 2 shows the five (5) different types of conditions for person count and how data should be collected properly. The guideline ensures the data collected is unbiased and the collection method is consistent.

The traffic count survey was conducted on weekdays and weekends for each site. Nevertheless, the timing and duration were subjected to the condition of each site. For example, shopping malls were observed to have heavy traffic after 10 pm during the preliminary site visits. Hence, the traffic survey was extended to 10:30 pm. Nevertheless, the data collected is sufficient to classify the timing into morning peak (before noon), afternoon peak (between 12 noon and 6 pm), and evening peak (after 6 pm).

### Vehicular trip, person trip, and equivalent vehicle trip

Most trip generation studies focused on the vehicular traffic volume without considering the person trip and pedestrian trip. These limitations have been highlighted in this study. Hence, this research included the person-trip by considering pedestrians entering and exiting the MXD site to address these limitations. The person-trip was calculated by summing the person inside the vehicle (driver and passenger) and pedestrian using Eq. (1). The person-trip for this study is defined as trip made by people, resident or shopper that entering and exiting the MXD premise, either walking or travelled with motorcars.

1 $${\text{Person Trip}} = {\text{Number of Person inside the Vehicle}} + {\text{Number of Pedestrian}}$$

As shown in Eqs. (2) and (3), the Vehicle to Person Ratio (VPR) for different vehicle classes must be calculated to determine the equivalent vehicular trip considering a person trip. VPR is defined as the ratio between total number of vehicle and person. The average VPR can be calculated by different vehicle class, such as motorcar, van, motorcycle, etc.

2 $${\text{Vehicle to Person Ratio }}\left( {{\text{VPR}}} \right) = \frac{{\text{Number of Vehicles}}}{{\text{Number of Persons}}}$$

3 $$\overline{{{\text{VPR}}}} = \frac{{\mathop \sum \nolimits_{{{\text{i}} = 1}}^{{\text{N}}} {\text{VPR}}}}{{\text{N}}}\quad {\text{For N types of vehicle class}}$$

Subsequently, the Vehicle Conversion Value (VCV) is selected by taking the average of VPR at different gates of traffic observation, or different entry points.

4 $${\text{Vehicle Conversion Value }}\left( {{\text{VCV}}} \right) = \frac{{\mathop \sum \nolimits_{{{\text{i}} = 1}}^{{\text{N}}} \overline{{{\text{VPR}}}} }}{{\text{N}}}\quad {\text{For N gates}}$$

For example, a VCV of 0.8 represents ten pedestrians or eight vehicles entering or exiting the MXD site. In other words, ten-person trips are equivalent to eight vehicular trips. The VCV is expected to be multiplied by the number of pedestrians to get the Equivalent Vehicle Number of Pedestrian (EVNP) (see Eq. (5). EVNP is defined as number of equivalent vehicular trips made by people that walking into or exit the MXD premise.

5 $${\text{Equivalent Vehicle Number of Pedestrian }}\left( {{\text{EVNP}}} \right) = {\text{VCV }} \times {\text{Number of Pedestrians}}$$

Finally, the actual vehicular trip, including the pedestrian count (Equivalent Vehicle Volume (EVV)), can be calculated by combining the existing vehicular trip with EVNP (see Eq. 6). The aim is to integrate person trips into the vehicular trips, so that the total vehicular trips are more practical.

6 $${\text{Equivalent Vehicle Volume}} = {\text{Existing Vehicular Trip}} + {\text{EVNP}}$$

### MXD’s passenger car unit (PCU)

The PCU is a measure in-vehicle unit to assess the highway capacity and represents the effects of changes in traffic composition. Different vehicles are assigned different values according to the space they take up. A passenger car has a value of 1. Smaller vehicles have lower values, while larger vehicles have high values. The PCU conversion factor at each MXD site was calculated using the MTGM methods. Table 2 shows the provisional PCU for each type of vehicle provided by MTGM. The provision served as an example of calculating the PCU conversion factor based on the vehicle composition. In the MTGM guideline, Car, Taxi, and Van were grouped with 1.00 provision PCU.

**Table 2:** Example of PCU conversion factor from MTGM^1^.

Access mode	Traffic percent	Provisional PCU	PCU conversion factor
Car/taxi	59.99	1.00	0.60
Motorcycle	30.45	0.33	0.10
Light lorry (2 axles)	7.83	1.75	0.14
Heavy lorry (> 2 axles)	0.31	2.25	0.01
Bus	1.42	2.25	0.03
Total	100.00		0.88

The PCU conversion factor was determined on different days of data collection, site, ingress, and egress. Eventually, the traffic volume in vehicles per hour obtained from the survey will be converted to PCU per hour based on the actual traffic composition on the MXD site.

### MXD trips generation

The trips generated for each site were estimated per MTGM 2010 based on two land uses, namely residential and commercial (retail shops or shopping malls). The commuter peak and generator peak, including morning peak (AM peak) and afternoon peak (PM peak) for each land use, were estimated using the equations recommended in MTGM. The trip generated was expressed in PCU per hour.

Commuter peak is defined as the period during the day when commuter traffic is highest. According to MTGM 2010, the morning peak has been defined as the period from 7:00 am to 10:00. The highest one-hour flow in the period is the AM Peak Hour of Commuter. The afternoon commuter peak period occurs between 4:00 to 7:00 pm, while the highest one-hour flow within the period is the PM Peak Hour of Commuter. These periods correspond to the time of the day when traffic flows on the street adjacent to a survey site are typically highest. The commuter peak is the best to apply for the trip estimation of residential and office land uses.

On the other hand, generator peak is defined as the highest one-hour of traffic flow during the day when traffic enters or exits a site. This hour may or may not correspond with the peak period of the adjacent street. The AM Peak Hour of Generator is the highest one-hour traffic generation for the proposed project before noon. In contrast, the PM Peak hour of the Generator is the highest one-hour of traffic generator in the afternoon. The generator peak is essential to estimate the trip generated by retail shops or shopping malls.

In order to be more conclusive and comparative with the actual traffic survey, both commuter and generator peaks during the morning and afternoon were calculated. The total trips by the two land uses were summed and tabulated as minimum, maximum, and average (middle of the range) values. These estimations were done for comparison and used for the development of the MXD adjustment factor.

## Results and discussion

### VCV and PCU conversion factor

Table 3 shows the computation outcomes of VCV and PCU conversion factors for the three different MXD sites. The VCV was applied to convert the number of pedestrians to obtain the EVNP expressed in the form of vehicles per hour. The PCU conversion factor was later used to convert the traffic volume as PCU per hour.

**Table 3:** VCV and PCU conversion factor for all MXD sites.

MXD Site	Vehicle conversion value		PCU conversion factor	
	Ingress (In)	Egress (Out)	Ingress (In)	Egress (Out)
Site A	0.762	0.808	0.99	0.99
Site B	0.628	0.629	0.98	0.99
Site C	0.733	0.707	0.96	0.96
Average	0.708	0.715	0.98	0.98
	0.711		0.98	

The VCV is valuable for future estimation, especially for MXD, which requires converting pedestrian numbers to vehicular trips (vehicle per hour). Although the VCV for overall average, ingress or egress are different, the average value of 0.711 will be selected as final VCV. To simplify subsequent calculation and discussion, the average value was rounded up to one significant figure and 0.7 will be used as final VCV instead, representing that those ten (10) pedestrians are equivalent to seven (7) vehicles entering or exiting the MXD premise. The VCV is also useful for future estimation of the modal split model (or mode choice model), especially on pedestrian changing their travel ways. The majority of modes of the transport entering and exiting the MXD premises were observed to be passenger cars. The evidence was found in the traffic composition for each site during traffic survey counts. Other types of vehicles, such as motorcycle, was minimum. Thus, the MXD attracted passenger cars the most. The motorcar ownership appears to be higher than motorcycles in the MXD premise. A typical transportation study always converts the vehicular trips in the vehicle per hour to PCU per hour. This conversion is to ensure the comparison are equal for different traffic compositions. Vehicle per hour might not reflect the actual situation of traffic scenario if a variation exists in the traffic composition.

### Traffic volume versus MTGM trip generated

Tables 4, 5, 6 shows the traffic volume (PCU/hr), MTGM Trip Generated (PCU/hr), and adjustment factor for each MXD site. The traffic volumes were collected from the actual traffic survey at different MXD sites, while the MTGM trip generated rates were estimated by using the manual. The MXD trip generation rates with MTGM were estimated by summing the trip generation rates of the two different single land-uses with appropriate parameters. The minimum and maximum of the traffic volume were observed during the morning or evening peak period, average values were obtained by averaging the minimum and maximum values. Meanwhile for MTGM trip generated rates, there were various of rates estimated with multiple models, the minimum, maximum and average values were selected from the outcomes of forecasting models in the manual. The vehicular and person trips were counted during the traffic survey and subsequently converted to EVV in the vehicle per hour and PCU per hour. The computation and conversion involved the application of VCV, EVNP, and PCU conversion factors. Nevertheless, few methods are available for trip generation rates to estimate the trip generation rates for individual land use as recommended in the MTGM. Additionally, MXD trip generation rates were determined by summing two single land use parameters’ values. Based on various models and methods in MTGM, the minimum and maximum values were tabulated accordingly. The average value was computed by averaging the minimum and maximum values, also considered the middle of the range.

**Table 4:** Traffic volume, MTGM trip generated, and adjustment factor at 1st MXD site (Site A).

	Minimum				Average				Maximum			
	Ingress (In)		Egress (Out)		Ingress (In)		Egress (Out)		Ingress (In)		Egress (Out)	
	AM Peak	PM Peak	AM Peak	PM Peak	AM Peak	PM Peak	AM Peak	PM Peak	AM Peak	PM Peak	AM Peak	PM Peak
Traffic volume (PCU/hr)	255	622	221	638	326	632	277	643	397	643	334	647
MTGM trip generated (PCU/hr)	220	515	562	429	509	843	578	693	899	1188	979	974
Difference	− 35	− 108	341	− 209	183	210	301	51	502	546	645	326
Adjustment factor	1.16	1.21	0.39	1.49	0.64	0.75	0.48	0.93	0.44	0.54	0.34	0.67

**Table 5:** Traffic volume, MTGM trip generated, and adjustment factor at 2nd MXD site (Site B).

	Minimum				Average				Maximum			
	Ingress (In)		Egress (Out)		Ingress (In)		Egress (Out)		Ingress (In)		Egress (Out)	
	AM peak	PM peak	AM peak	PM peak	AM peak	PM peak	AM peak	PM peak	AM peak	PM peak	AM peak	PM peak
Traffic volume (PCU/hr)	60	110	102	117	78	125	105	122	96	141	109	126
MTGM trip generated (PCU/hr)	71	161	184	125	143	248	197	195	239	340	302	270
Difference	11	51	82	8	65	123	91	74	144	199	193	144
Adjustment factor	0.85	0.68	0.55	0.94	0.54	0.50	0.54	0.62	0.40	0.41	0.36	0.47

**Table 6:** Traffic volume, MTGM trip generated, and adjustment factor at 3^rd^ MXD site (Site C).

	Minimum				Average				Maximum			
	Ingress (In)		Egress (Out)		Ingress (In)		Egress (Out)		Ingress (In)		Egress (Out)	
	AM peak	PM peak	AM peak	PM peak	AM peak	PM peak	AM peak	PM peak	AM peak	PM peak	AM peak	PM peak
Traffic volume (PCU/hr)	142	238	225	194	160	277	296	208	179	315	367	222
MTGM trip generated (PCU/hr)	168	294	371	208	239	430	491	313	301	568	642	425
Difference	26	56	147	14	79	154	195	105	122	252	275	203
Adjustment factor	0.84	0.81	0.61	0.93	0.67	0.64	0.60	0.67	0.59	0.56	0.57	0.52

The MXDs adjustment factor is summarised in Table 7. In general, the trip generation rates at Site A were observed to be underestimated if minimum values were used to compare with the traffic survey count. Contrarily, Site B and C were considered overestimated. Nevertheless, this variation is not a primary concern in the real application as none of the minimum values is adopted to present the traffic scenario. Overall, the average and maximum values showed overestimated trip-generated rates. The adoption of average value is common for traffic engineers in the industry. Hence, by observing the average values, the trip generated for MXD was found to be overestimated and should be reduced.

**Table 7:** MXDs adjustment factor.

MXD Site	Minimum				Average				Maximum			
	Ingress (In)		Egress (Out)		Ingress (In)		Egress (Out)		Ingress (In)		Egress (Out)	
	AM peak	PM peak	AM peak	PM peak	AM peak	PM peak	AM peak	PM peak	AM peak	PM peak	AM peak	PM peak
Site A	1.16	1.21	0.39	1.49	0.64	0.75	0.48	0.93	0.44	0.54	0.34	0.67
Site B	0.85	0.68	0.55	0.94	0.54	0.50	0.54	0.62	0.40	0.41	0.36	0.47
Site C	0.84	0.81	0.61	0.93	0.67	0.64	0.60	0.67	0.59	0.56	0.57	0.52
Average	0.95	0.90	0.52	1.12	0.62	0.63	0.54	0.74	0.48	0.50	0.42	0.55

The comparison between the traffic survey counts and MTGM trip generated rates has shown significant evidence to prove that the rates were overestimated. This conclusion was made by comparing the average values of the differences in each site where most were overestimated. One of the approaches to estimate highly reliable trip generation rates were to formulate the adjustment factor, which can be applied concurrently with MTGM. The adjustment factors were computed by dividing the traffic survey count by trip generation rates in different scenarios. Ideally, the adjustment factor can be applied by multiplying the MTGM trip generation rate for a newly proposed MXD.

The recommended adjustment factor for Ingress AM, Ingress PM, Egress AM, and Egress PM Peaks are 0.62, 0.63, 0.54, and 0.74, respectively. Users first need to estimate the total generation trips using MTGM by summing two different land use to apply these adjustment factors. One land use must be residential, while another should be a shopping mall or retail shop. The MTGM trip generation rates will be reduced by multiplying the respective adjustment factor. These guidelines will simplify the process while ensuring that future trips are not overestimated, leading to a high cost for upgrading the surrounding transport infrastructure.

### Validation of MXD adjustment factor

The objective of the adjustment factor is to improve the MXD trip generation rates by reducing the forecasted value obtained from MTGM. A traffic survey was conducted at the fourth site (Site D) to validate the recommended adjustment factor presented in Table 8. Similar to the other MXD sites, the minimum, maximum and average of the traffic volumes were observed during the peak periods. The traffic survey conducted on both weekdays and weekends revealed that actual traffic volumes (Veh/hr and PCU/hr) were lower than the MTGM forecasted values. This traffic survey was conducted after the Movement Control Order (MCO) was lifted in Klang Valley, Malaysia. The MCO started on March 2020 due to COVID-19 pandemic. This study actually was interrupted by national lockdown for two years. The traffic surveys for first three MXD sites were conducted prior the MCO and the fourth site only can be continued after the MCO. Nevertheless, the observed period of fourth site may not fully represent the normal or full capacity of the actual traffic flow and possibly represent only 50% of the actual traffic pattern. Although the adjustment factors were established using the traffic data before MCO, the trend remains unchanged. The forecasted MTGM values are still considered to be extremely high. For example, as shown in Table 8, by magnifying the survey data to simulate the full capacity during the normal situation and assuming an additional 50% traffic volume, the MTGM trip generated is still considered overestimated.

**Table 8:** Traffic volume at the 4th MXD site (Site D) and comparison with an adjustment factor.

	Weekday				Weekend			
	Ingress (In)		Egress (Out)		Ingress (In)		Egress (Out)	
	AM peak	PM peak	AM peak	PM peak	AM peak	PM peak	AM peak	PM peak
Traffic volume (Veh/hr)	272	378	204	414	450	502	300	557
Traffic volume (PCU/hr)	274	378	206	411	446	499	301	549
Traffic volume (PCU/hr) by simulating to normal situation	548	756	412	822	892	998	602	1098
MTGM trip generated (PCU/hr)	789	1745	517	1649	1938	2433	1556	2050
MTGM trip generated with adjustment factor (PCU/hr)	489	1099	279	1220	1202	1533	840	1517

With the application of the adjustment factor, the improved MTGM trip generation rates were observed to be closer to the actual traffic survey overall. Adjustment factors should be applied to discount the MTGM trip generation rates to avoid overspending and undertaking too many efforts to upgrade the road infrastructure.

## Conclusion

This research requires a new approach for data collection, which should consider person trips for the MXD trip generation trips in PCU per hour. Hence, a new methodology has been formulated to capture the pedestrians’ movement, either walking or exiting the MXD premises, in the morning, afternoon, and evening. A new procedure has been introduced and applied in this research. Besides, guidelines to collect pedestrian or person-trips have been established. The study intended to standardise the pedestrian count to ensure that the data collected was more reliable and avoided miscommunication among traffic enumerators.

Secondly, the VCV is recommended as 0.7, representing that ten (10) pedestrians are equivalent to seven (7) vehicles entering or exiting the MXD premise with two land uses. This value can be used for estimation by converting the vehicular trip to a person-trip or vice versa. Third, the PCU conversion factors calculated from the MXD sites were highly consistent and had minimal variations. The values indicated that the motorcar or passenger car volume was the highest compared to other types of vehicles. Hence, the PCU conversion unit for the MXD site is recommended as 0.98, where the application is to simply multiply with vehicular trips (vehicle per hour) to be converted to PCU per hour for an MXD site.

Moreover, the idea of introducing the MXD adjustment factor was to reduce the MTGM trip generation rates. The current practice of estimating the MXD trip generation rates is simply by summing multiple single land use. Such practice was an overestimating approach, which may cause the overdesign of transportation infrastructure. The adjustment factor was determined using the peak hour traffic survey count (PCU/hr) and dividing the MTGM trip generation rates (PCU/hr) at the peak hour. Table 7 shows that there is not much variation in ingress and egress traffic movement. Hence, a single MXD adjustment factor was established, which is 0.63. The application of this adjustment factor is effortless. The user needs first to compute the MXD trip generation rates using the MTGM and subsequently multiple the adjustment factor in reducing or discounting the forecasted rates. Nevertheless, this factor only can be applied to two land uses, comprising residential and retail shops or shopping malls. This approach was considered as a supplement guideline alongside MTGM.

Overestimating the trip generation rates for MXD will cause overly investment and construction on road infrastructure, this is mainly due to overestimating the traffic demands. For example, low traffic intersection to be converted as signalised intersection due to overestimation may resulting significant traffic delay and congestion. By using this adjustment approach, the estimation of traffic demand will be more practical, and the reduction of forecasted rates can be justified. Currently the findings were limited to three selected MXD site, on two different land-use. This study can be enhanced with more MXD sites that have more than two different land-use. Secondly the traffic survey should be conducted after MCO whereby the traffic condition is more stable and mature.

## Supplementary Information

Supplementary Information.

## Data Availability

The datasets generated during the current study are available in the Raw Traffic Survey Data repository.
